# New Coleoptera records from New Brunswick, Canada: Mycetophagidae, Tetratomidae, and Melandryidae

**DOI:** 10.3897/zookeys.179.2598

**Published:** 2012-04-04

**Authors:** Reginald P. Webster, Jon D. Sweeney, Ian DeMerchant

**Affiliations:** 1 Natural Resources Canada, Canadian Forest Service - Atlantic Forestry Centre, 1350 Regent St., P.O. Box 4000, Fredericton, NB, Canada E3B 5P7

**Keywords:** Mycetophagidae, Tetratomidae, Melandryidae, new records, Canada, New Brunswick

## Abstract

We report 21 new species records for the Coleoptera fauna of New Brunswick, Canada, seven of which are new records for the Maritime provinces. Four species of Mycetophagidae (*Litargus didesmus* Say, *Litargus tetrapilotus* LeConte, *Mycetophagus punctatus* Say, and *Mycetophagus quadriguttatus* Müller) are newly reported for the province of New Brunswick. *Litargus didesmus* is newly recorded for the Maritime provinces. Seven species of Tetratomidae are added to the faunal list of New Brunswick: *Eustrophus tomentosus* Say, *Penthe obliquata* (Fabricius), and *Tetratoma tessellata* Melsheimer are new to New Brunswick: *Hallomenus serricornis* LeConte, *Pisenus humeralis* Kirby, *Synstrophus repandus* (Horn), and *Tetratoma variegata* Casey, which are newly recorded for New Brunswick and the Maritime provinces. Ten additional species of Melandryidae are reported from New Brunswick, of which *Orchesia cultriformis* Laliberté, *Orchesia ovata* Laliberté, *Phloeotrya fusca* (LeConte), *Scotochroides antennatus* Mank, *Spilotus quadripustulatus* (Melsheimer), *Symphora flavicollis* (Haldeman), *Symphora rugosa* (Haldeman), and *Zilora hispida* LeConte are new for the province, and *Microscapha clavicornis* LeConte and *Zilora nuda* Provancher are newly recorded for the Maritime provinces. In addition, we report numerous additional records for three species of Mycetophagidae and one species of Melandryidae previously recorded from New Brunswick that suggest these species are more widely distributed than previously known. Collection, habitat data, and distribution maps are presented for all these species.

## Introduction

The Melandryidae and Tetratomidae of the Maritime provinces of Canada were reviewed by Majka and Pollock (2006). More recently, Majka (2010) reviewed the Mycetophagidae of the region. Intensive collecting in New Brunswick by the first author since 2003 and records obtained from by-catch samples during a study to develop a general attractant for the detection of invasive species of Cerambycidae have yielded additional new provincial records in the above families. The purpose of this paper is to report on these new records. A brief synopsis of each family is included in the results below.

## Methods and conventions

The following records are based on specimens collected during a general survey by the first author to document the Coleoptera fauna of New Brunswick and from by-catch samples obtained in Lindgren 12-funnel traps placed at various sites in New Brunswick from 2008–2011 as part of a study to develop improved lures for survey of potentially invasive species of Cerambycidae. Additional records were obtained from specimens contained in the collection belonging to Natural Resources Canada, Canadian Forest Service - Atlantic Forestry Centre, Fredericton, New Brunswick.

## Collection methods

Various methods were employed to collect the species reported in this study. Details are outlined in Webster et al. (2009, Appendix). Many specimens were collected in Lindgren funnel traps. These traps mimic tree trunks and are often effective for sampling species of Coleoptera that live in microhabitats associated with standing trees (Lindgren 1983). See Webster et al. (in press) for details of the methods used for deployment of Lindgren 12-funnel traps and sample collection. A description of the habitat was recorded for all specimens collected during this survey. Locality and habitat data are presented exactly as on labels for each record. This information, as well as additional collecting notes, is summarized and discussed in collection and habitat data for each species.

## Distribution

Distribution maps, created using ArcMap and ArcGIS, are presented for each species in New Brunswick. Every species is cited with current distribution in Canada and Alaska, using abbreviations for the state, provinces, and territories. New records for New Brunswick are indicated in bold under Distribution in Canada and Alaska. The following abbreviations are used in the text:

**Table:** 

**AK**	Alaska	**MB**	Manitoba
**YT**	Yukon Territory	**ON**	Ontario
**NT**	Northwest Territories	**QC**	Quebec
**NU**	Nunavut	**NB**	New Brunswick
**BC**	British Columbia	**PE**	Prince Edward Island
**AB**	Alberta	**NS**	Nova Scotia
**SK**	Saskatchewan	**NF & LB**	Newfoundland and Labrador*

*Newfoundland and Labrador are each treated separately under the current Distribution in Canada and Alaska.

Acronyms of collections examined or where specimens reside referred to in this study are as follows:

**AFC** Atlantic Forestry Centre, Natural Resources Canada, Canadian Forest Service, Fredericton, New Brunswick, Canada

**CNC** Canadian National Collection of Insects, Arachnids and Nematodes, Agriculture and Agri-Food Canada, Ottawa, Ontario, Canada

**NBM** New Brunswick Museum, Saint John, New Brunswick, Canada

**RWC** Reginald P. Webster Collection, Charters Settlement, New Brunswick, Canada

## Results

### Species accounts

All records below are species newly recorded for New Brunswick, Canada, unless noted otherwise (additional records). Species followed by ** are newly recorded from the Maritime provinces (New Brunswick, Nova Scotia, Prince Edward Island) of Canada.

The classification of the Mycetophagidae, Tetratomidae, and Melandryidae follows Bouchard et al. (2011).

### Family Mycetophagidae Leach, 1815

The Mycetophagidae (the hairy fungus beetles) are found in mushrooms or fleshy polypore fungi that have begun to dehydrate, under fungus-covered bark or on moldy vegetative material (Young 2002). Some species, such as *Typhaea stercorea* (Linnaeus), are often found in stored products. Majka (2010) reviewed the Mycetophagidae of the Maritime provinces and reported four species for New Brunswick, three for the first time. Four additional species (*Mycetophagus punctatus* Say, *Mycetophagus quadriguttatus* Müller, *Litargus didesmus* Say, and *Litargus tetrapilotus* LeConte) are reported here from the province, as well as new localities and additional bionomic data for the three species recently reported by Majka (2010) (Table 1). *Litargus didesmus* is newly recorded for the Maritime provinces.

**Table 1.** Species of Mycetophagidae, Tetratomidae, and Melandryidae reported from New Brunswick, Canada.

**Family Mycetophagidae Leach**
**Subfamily Mycetophaginae Leach**
**Tribe Mycetophagini Leach**
*Mycetophagus flexuosus* Say
*Mycetophagus punctatus* Say*
*Mycetophagus serrulatus* Casey
*Mycetophagus pluripunctatus* LeConte
*Mycetophagus quadriguttatus* Müller*
**Tribe Typhaeini Thomson**
*Typhaea stercorea* (Linnaeus)
*Litargus didesmus* Say**
*Litargus tetraspilotus* LeConte*
**Family Tetratomidae Billberg**
**Subfamily Tetratominae Billberg**
*Tetratoma tessellata* Melsheimer*
*Tetratoma variegata* Casey**
**Subfamily Piseninae Miyatake**
*Pisenus humeralis* Kirby**
**Subfamily Penthinae Lacordaire**
*Penthe obliquata* (Fabricius)*
*Penthe pimelia* (Fabricius)
**Subfamily Hallomeninae Gistel**
*Hallomenus serricornis* LeConte**
**Subfamily Eustrophinae Gistel**
**Tribe Eustrophini Gistel**
*Eustrophus tomentosus* Say*
*Synstrophus repandus* (Horn)**
**Tribe Holostrophini Nikitsky**
*Pseudoholostrophus discolor* (Horn)
**Family Melandryidae Leach**
**Subfamily Melandryidae Leach**
**Tribe Dircaeni Kirby**
*Dircaea liturata* (LeConte)
**Tribe Hypulini Gistel**
*Hypulus simulator* Newman
*Symphora flavicollis* (Haldeman)*
*Symphora rugosa* (Haldeman)*
**Tribe Melandryini Leach**
*Emmesa connectens* Newman
*Emmesa labiata* (Say)
*Melandrya striata* Say
*Phryganophilus collaris* LeConte
*Prothalpia undata* LeConte
**Tribe Orchesiini Mulsant**
*Microscapha clavicornis* LeConte**
*Orchesia castanea* (Melsheimer)
*Orchesia cultriformis* Laliberté*
*Orchesia ovata* Laliberté*
**Tribe Serropalpini Latreille**
*Enchodes sericea* (Haldeman)
*Scotochroa atra* LeConte
*Scotochroa buprestoides* (Kirby)
*Scotochroides antennatus* Mank*
*Serropalpus coxalis* Mank
*Serropalpus substriatus* Haldeman
*Phloeotyra fusca* (LeConte)*
*Spilotus quadripustulatus* (Melsheimer)*
*Xylita livida* (Sahlberge)
*Xylita laevigata* (Hellenius)
**Tribe Zilorini Desbrochers des Loges**
*Zilora hispida* LeConte*
*Zilora nuda* Provancher**

**Notes:** *New to province, **New to Maritime provinces.

### Subfamily Mycetophaginae Leach, 1815

**Tribe Mycetophagini Leach, 1815**

#### Mycetophagus flexuosus

Say, 1826

http://species-id.net/wiki/Mycetophagus_flexuosus

Map 1

##### Material examined. 

**Additional New Brunswick records, Carleton Co.**, Meduxnekeag Valley Nature Preserve, 46.1907°N, 67.6740°W, 8.VIII.2006, R. P. Webster, mature mixed forest, on partially dried *Pleurotus* species on dead standing sugar maple (1, RWC); Jackson Falls, Bell Forest, 46.2200°N, 67.7231°W, 26.VI.2007, R. P. Webster, mature hardwood forest, u.v. light (1, RWC); same locality, collector, and forest type, 9.VIII.2005, 13.VIII.2007, on partially dried *Pleurotus* species on dead standing sugar maple (1, RWC); same locality, collector, and forest type, 4–12.VI.2008, Lindgren funnel trap (1, AFC). **Queens Co.**, Grand Lake near Scotchtown, 45.8762°N, 66.1816°W, 19.IX.2006, R. P. Webster, oak and maple forest, in decayed log covered with gilled mushrooms and polypore fungi (1, RWC); Cranberry Lake P.N.A (Protected Natural Area), 46.1125°N, 65.6075°W, 24.IV-5.V.2009, 12–21.V.2009, R. Webster & M.-A. Giguère, old red oak forest, Lindgren funnel traps (2, AFC). **York Co.**, Charters Settlement, 45.8395°N, 66.7391°W, 9.VII.2006, R. P. Webster, mixed forest, u.v. light (1 RWC); same locality, collector, and forest type, 29.VIII.2007, 21.IX.2007, in pile of moldy corncobs and cornhusks (2, RWC); same locality and collector but 45.8340°N, 66.7450°W, 11.VII.2006, mature mixed forest, on partially dried *Pleurotus* species on dead standing trembling aspen (1, RWC); 15 km W of Tracy off Rt. 645, 45.6848°N, 66.8821°W, 1–8.VI.2009, R. Webster & M.-A. Giguère, old red pine forest, Lindgren funnel trap (1, AFC); same locality and habitat data but 18.V-2.VI.2010, R. Webster & C. MacKay, Lindgren funnel trap (1, AFC).

**Map 1.**
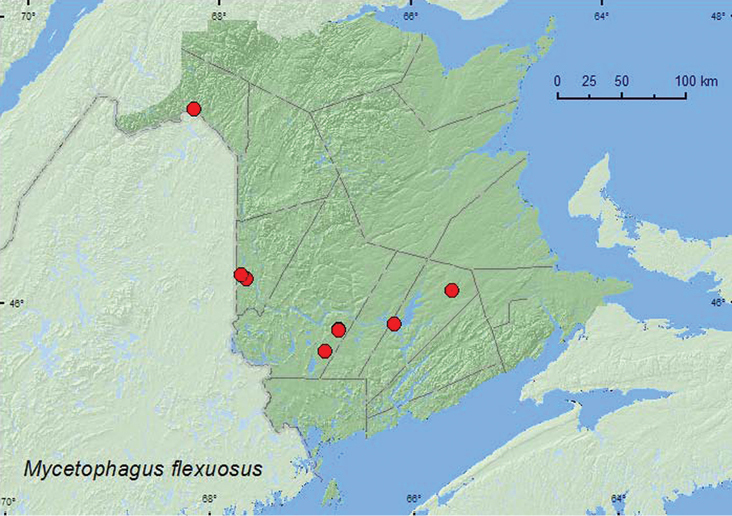
Collection localities in New Brunswick, Canada of *Mycetophagus flexuosus*.

##### Collection and habitat data.

Adults of *Mycetophagus flexuosus* in New Brunswick were found in hardwood forests with sugar maple (*Acer saccharum* Marsh.) and American beech (*Fagus grandifolia* Ehrh.), an old red oak (*Quercus rubra* L.) forest, mixed forests, and an old (180-year-old) red pine (*Pinus resinosa* Ait.) forest. This species was found in partially dried *Pleurotus* species on dead, standing sugar maples, on a dead, standing trembling aspen (*Populus tremuloides* Michx.), in a decayed log covered with gilled mushrooms and polypore fungi, in a pile of moldy corncobs and cornhusks, and at an ultraviolet light. Specimens were also captured in Lindgren funnel traps at several localities. Cline and Leschen (2005) reported *Mycetophagus flexuosus* from the oyster mushroom, *Pleurotus ostreatus* Fries. Other fungal associations with this species were reported in Majka (2010). Adults in New Brunswick were collected during April, May, June, July, August, and September.

##### Distribution in Canada and Alaska.

MB, ON, QC, NB (Bousquet 1991; Majka 2010). Makja (2010) reported this species for the first time from New Brunswick and the Maritime provinces based on two specimens collected in Edmundston (Madawaska Co.) by Richard Migneault. The above records indicate that this species in not uncommon and is probably widespread in New Brunswick.

#### Mycetophagus punctatus

Say, 1826

http://species-id.net/wiki/Mycetophagus_punctatus

Map 2

##### Material examined.

**New Brunswick, Carleton Co.**, Meduxnekeag Valley Nature Preserve, 46.1883°N, 67.6745°W, 9.VIII.2005, R. P. Webster & M.-A. Giguère, mature hardwood forest, on partially dried *Pleurotus* species on dead standing sugar maple (3, RWC); Jackson Falls, Bell Forest, 46.2200°N, 67.7215°W, 9.VIII.2005, R. P. Webster & M.-A. Giguère, mature hardwood forest, on partially dried *Pleurotus* species on dead standing sugar maple (6, RWC); same locality but 46.2200°N, 67.7231°W, 19–27.VI.2008, R. P. Webster, mature hardwood forest, Lindgren funnel trap (1, AFC). **Sunbury Co.**, Burton near Sunpoke Lake, 45.7658°N, 66.5546°W, 20.VI.2007, R. P. Webster, red oak and red maple forest, on slightly dried *Pleurotus ostreatus* on dead standing poplar (1, RWC). **York Co.**, Canterbury, near Browns Mountain Fen, 45.8876°N, 67.6560°W, 3.VIII.2006, R. Webster & M.-A. Giguère, mature hardwood forest, on partially dried *Pleurotus* species on dead standing sugar maple (1, NBM).

**Map 2.**
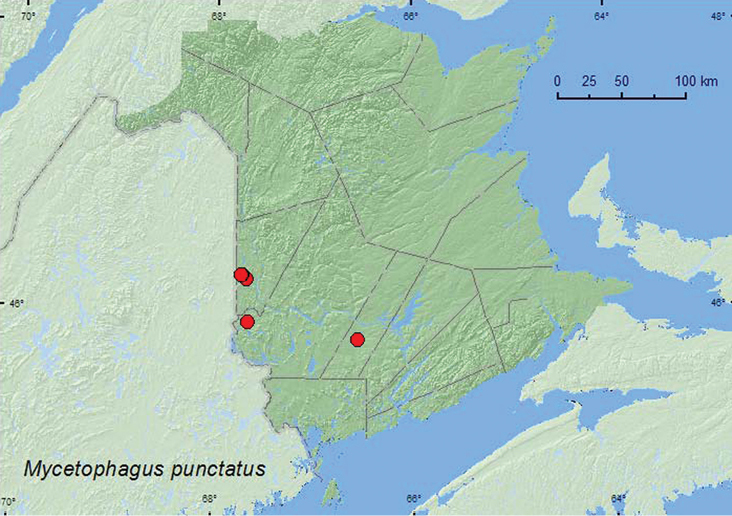
Collection localities in New Brunswick, Canada of *Mycetophagus punctatus*.

##### Collection and habitat data.

All the records of this species from New Brunswick were from hardwood forests (sugar maple and American beech, red oak and red maple (*Acer rubrum* L.)). Most adults were collected from partially dried *Pleurotus* species, including the oyster mushroom, *Pleurotus ostreatus*, on dead, standing sugar maples and a dead, standing poplar (probably trembling aspen). One individual was captured in a Lindgren funnel trap. Cline and Leschen (2005) also reported *Mycetophagus punctatus* from *Pleurotus ostreatus*. Majka (2010) reported other fungal associations with this species. Adults were captured during June and August.

##### Distribution in Canada and Alaska.

MB, ON, QC, **NB**, NS (Bousquet 1991; Majka 2010). Majka (2010) newly recorded this species from Nova Scotia and the Maritime provinces.

#### Mycetophagus serrulatus

Casey, 1900

http://species-id.net/wiki/Mycetophagus_serrulatus

Map 3

##### Material examined.

**Additional New Brunswick records. Carleton Co.**, Meduxnekeag Valley Nature Preserve, 46.1907°N, 67.6740°W, 20.VI.2006, R. P. Webster, mature mixed forest, on partially dried *Pleurotus* species on dead standing trembling aspen (1, RWC); same locality but 46.1877°N, 67.6717°W, 2.IX.2008, R. P. Webster, mature hardwood forest, on slightly dried *Climacodon septentrionale* on sugar maple (9, NBM, RWC); Jackson Falls, Bell Forest, 46.2200°N, 67.7231°W, 7.VI.2007, R. P. Webster, mature hardwood forest, in polypore fungi on large basswood log (1, NBM); same locality and forest type but 20–26.V.2009, R. Webster & M.-A. Giguère, Lindgren funnel trap (1, AFC). **Queens Co.**, Cranberry Lake P.N.A, 46.1125°N, 65.6075°W, 5–11.VI.2009, R. Webster & M.-A. Giguère, mature red oak forest, Lindgren funnel trap (1, AFC). **York Co.** Canterbury, near Browns Mountain Fen, 45.8876°N, 67.6560°W, 3.VIII.2006, mature hardwood forest, on partially dried *Pleurotus* species on dead standing sugar maple (2, RWC); Charters Settlement, 45.8395°N, 66.7391°W, 20.VII.2006, R. P. Webster, mixed forest, u.v. light (1, RWC).

**Map 3.**
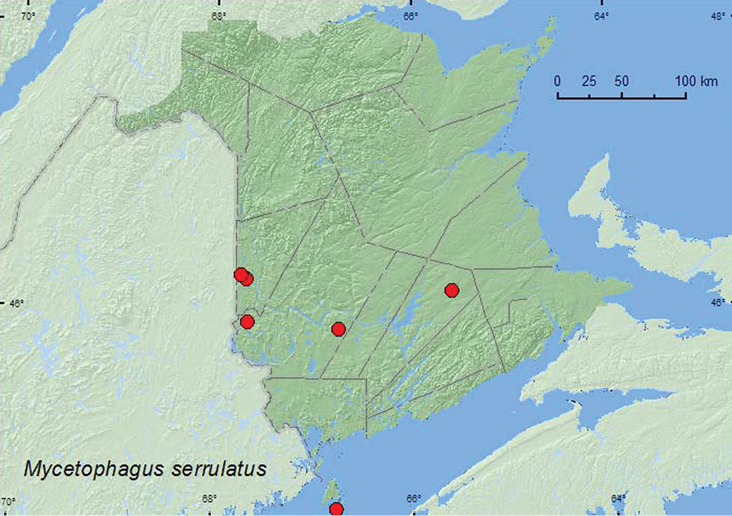
Collection localities in New Brunswick, Canada of *Mycetophagus serrulatus*.

##### Collection and habitat data.

Most adults of *Mycetophagus serrulatus* from New Brunswick were collected in hardwood forests (sugar maple and American beech, red oak) or mixed forests. Majka (2010) reported a specimen from a balsam fir (*Abies balsamea* (L.) Mill.) forest. Adults were found on slightly dried *Climacodon septentrionale* (Fr.) Karsten on sugar maple, in polypore fungi on a large basswood (*Tilia americana* L.) log, and in partially dried *Pleurotus* sp. on dead, standing sugar maples and trembling aspen. Adults were collected during June, July, August, and September.

##### Distribution in Canada and Alaska.

MB, ON, QC, NB, NS (Bousquet 1991; Dollin et al. 2008; Majka 2010). Majka (2010) newly reported this species from New Brunswick from the Grand Manan archipelago, Kent Island (Charlotte Co.). This species is probably widespread in the province.

#### Mycetophagus pluripunctatus

LeConte, 1856

http://species-id.net/wiki/Mycetophagus_pluripunctatus

Map 4

##### Material examined.

**Additional New Brunswick records. Carleton Co.**, Jackson Falls, Bell Forest, 46.2200°N, 67.7231°W, 6.V.2007, R. P. Webster, mature hardwood forest, on fleshy polypore (bracket) fungi on dead standing beech (5, NBM, RWC); Belleville, 1.3 km E jct. Rt. 540 and Plymouth Rd., 46.1867°N, 67.6817°W, 7.V.2008, R. P. Webster, old hardwood forest, on fleshy (shelf) polypore fungi on beech log (2, RWC). **Queens Co.**, Cranberry Lake P.N.A, 46.1125°N, 65.6075°W, 24.IV-5.V.2009, 27.V–5.VI.2009, 5–11.VI.2009, R. Webster & M.-A. Giguère, mature red oak forest, Lindgren funnel traps (5, AFC). **Restigouche Co.**, vic. Summit Depot, 47.7836°N, 68.3227°W, 21.VII.2010, R. Webster and M. Turgeon, clear-cut, in decaying *Climacodon septentrionale* on dead standing yellow birch (1, RWC); Dionne Brook P.N.A., 47.9030°N, 68.3503°W, 30.V–15.VI.2011, M. Roy & V. Webster, old-growth northern hardwood forest, Lindgren funnel trap (1, NBM); same locality and collectors but 47.9064°N, 68.3441°W, 27.VI–14.VII.2011, old-growth white spruce and balsam fir forest, Lindgren funnel trap (1, AFC). **Sunbury Co.**, Acadia Research Forest, 45.9866°N, 66.3841°W, 16–24.VI.2009, R. Webster & M.-A. Giguère, mature (110-year-old) red spruce forest with scattered red maple and balsam fir, Lindgren funnel trap (1, AFC). **York Co.**, Canterbury, near Browns Mountain Fen, 45.8876°N, 67.6560°W, 3.VIII.2006, R. P. Webster, mature hardwood forest, on partially dried *Pleurotus* species on dead standing sugar maple (1, RWC); 15 km W of Tracy off Rt. 645, 45.6848°N, 66.8821°W, 25.IV–4.V.2009, R. Webster & M.-A. Giguère, old red pine forest, Lindgren funnel trap (1, AFC); same locality and habitat data but 18.V-2.VI.2010, R. Webster & C. MacKay, Lindgren funnel trap (1, AFC).

**Map 4.**
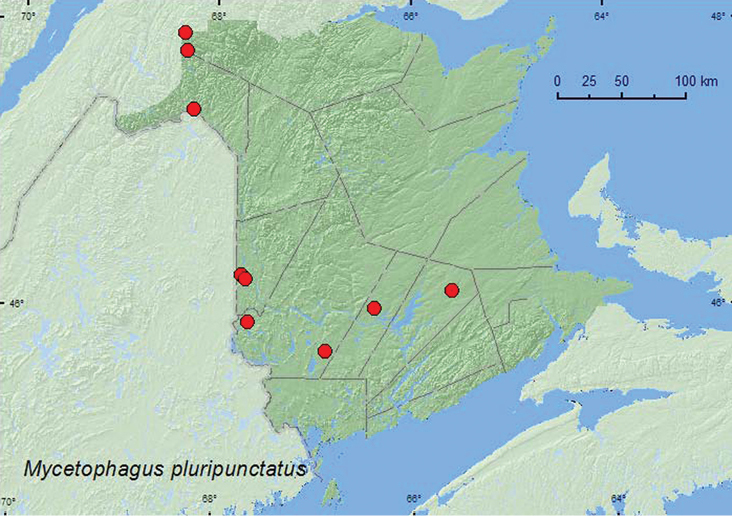
Collection localities in New Brunswick, Canada of *Mycetophagus pluripunctatus*.

##### Collection and habitat data.

*Mycetophagus pluripunctatus*was found in mature and old hardwood forests with sugar maple, American beech, white ash, and butternut (*Juglans cinerea* L.), an old-growth northern hardwood forest with sugar maple and yellow birch (*Betula alleghaniensis* Britt.), an old red oak forest, a mature (110-year-old) red spruce forest (*Picea rubens* Sarg.), an old-growth white spruce (*Picea glauca* (Moench) Voss) and balsam fir forest, and an old red pine forest. Adults were found in or on fleshy (bracket, shelf) polypore fungi on standing, dead American beech trees and logs, and on partially dried *Pleurotus* sp. on a dead, standing sugar maple. One individual was collected from a decaying *Climacodon septentrionale* on dead, standing yellow birch in a clearcut. Majka (2010) reported other fungal associations with this species. Adults were also captured in Lindgren funnel traps at several sites in New Brunswick. Adults were captured during April, May, June, July, and August.

##### Distribution in Canada and Alaska.

AB, MB, ON, QC, NB, NS (Bousquet 1991; Bishop et al. 2009; Majka 2010). Majka (2010) newly recorded this species from New Brunswick based on a specimen collected by R. Migneault in Edmundston (Madawaska Co.). This species is widespread in the province.

#### Mycetophagus quadriguttatus

Müller, 1821

http://species-id.net/wiki/Mycetophagus_quadriguttatus

Map 5

##### Material examined.

**New Brunswick, Carleton Co.**, Jackson Falls, Bell Forest, 46.2200°N, 67.7231°W, 4–12.VI.2008, R. P. Webster, mature hardwood forest, Lindgren funnel trap (1, AFC). **Queens Co.**, Cranberry Lake P.N.A., 46.1125°N, 65.6075°W, 7–22.VI.2011, M. Roy & V. Webster, old red oak forest, Lindgren funnel trap (1, RWC). **York Co.**, Charters Settlement, 45.8395°N, 66.7391°W, 5.IX.2006, 28.IX.2006, 29.VIII.2007, 21.IX.2007, 30.IX.2007, R. P. Webster, mixed forest, in decaying (moldy) corncobs and cornhusks (5, RWC).

**Map 5.**
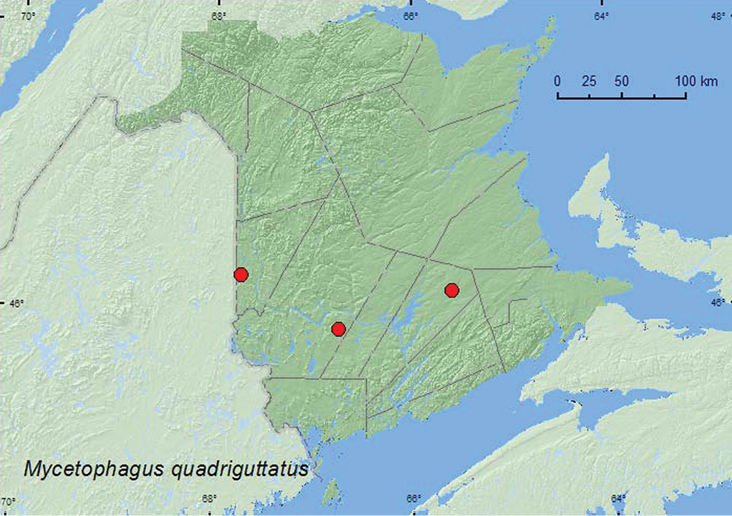
Collection localities in New Brunswick, Canada of *Mycetophagus quadriguttatus*.

##### Collection and habitat data.

Most adults from New Brunswick were collected from moldy decaying corncobs and cornhusks near a mixed forest. One individual each was captured in a Lindgren funnel trap deployed in a mature hardwood forest and an old red oak forest. Adults were collected during June, August, and September.

##### Distribution in Canada and Alaska.

BC, AB, SK, MB, ON, QC, **NB**, NS (Bousquet 1991; Majka 2010). Although *Mycetophagus quadriguttatus* was reported as occurring in New Brunswick by Bousquet (1991), no specimens could be located to support this record according to Majka (2010). In addition, Campbell et al. (1989) did not report it for the province, and thus, Majka considered the record as provisional. However, the above records establish this species as a member of the New Brunswick Coleoptera fauna.

### Tribe Typhaeini Thomson, 1863

#### Litargus didesmus

Say**

http://species-id.net/wiki/Litargus_didesmus

Map 6

##### Material examined.

**New Brunswick, Sunbury Co.**, Acadia Research Forest, 45.9816°N, 66.3374°W, 18.VI.2007, R. P. Webster, 8.5-year-old regenerating mixed forest (off Rd. 7), in gilled mushrooms on sun-exposed stump (8, NBM, RWC); same locality and collector but 46.0173°N, 66.3741°W, 18.VI.2007, 8.5-year-old regenerating mixed forest (off Rd. 16), in gilled mushrooms on sun-exposed stump (4, NBM, RWC); Burton, near Sunpoke Lake, 45.7658°N, 66.5546°W, 20.VI.2007, R. P. Webster, oak forest, on partially dried *Pleurotus* sp. on dead trembling aspen (1, RWC). **York Co.**, Charters Settlement, 45.8395°N, 66.7391°W, 27.VII.2004, R. P. Webster, mixed forest, at m.v. light (1, RWC).

**Map 6.**
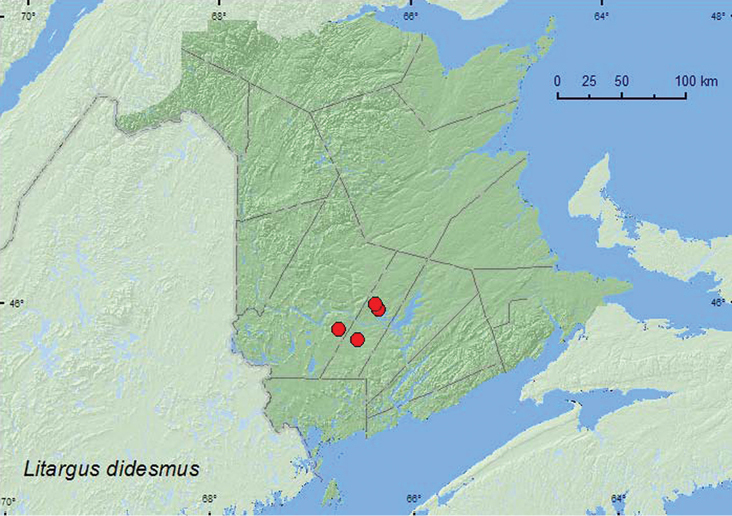
Collection localities in New Brunswick, Canada of *Litargus didesmus*.

##### Collection and habitat data.

This species was found in 8.5-year-old regenerating mixed forests, a mixed forest, and in a red oak stand. Adults were collected from gilled mushrooms on sun-exposed stumps, a group of partially dried *Pleurotus* sp. on a dead, standing trembling aspen, and at a mercury-vapor light. Adults were captured during June and July.

##### Distribution in Canada and Alaska.

QC, **NB** (Bousquet 1991).

#### Litargus tetraspilotus

LeConte, 1856

http://species-id.net/wiki/Litargus_tetraspilotus

Map 7

##### Material examined.

**New Brunswick, Queens Co.**, Cranberry Lake P.N.A., 46.1125°N, 65.6075°W, 18-31.VIII.2011, C. Hughes & R. P. Webster, old red oak forest, Lindgren funnel trap (1, RWC). **Restigouche Co.**, Jacquet River Gorge P.N.A., 47.7762°N, 66.1271°W, 18.VIII.2010, R. P. Webster, pine/spruce slope above Jacquet River, in decaying mushrooms (1, AFC).

**Map 7.**
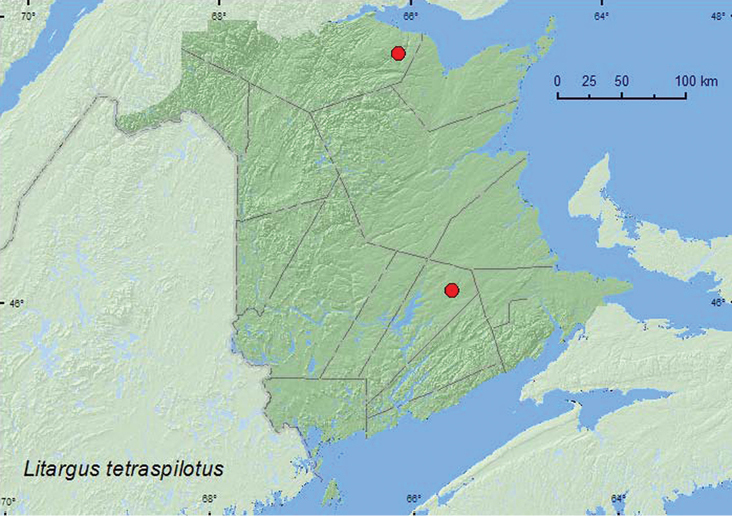
Collection localities in New Brunswick, Canada of *Litargus tetraspilotus*.

##### Collection and habitat data.

Majka (2010) reported *Litargus tetraspilotus* from deciduous, coniferous, and mixed forests, seashores, coastal and sandy pine barrens, and old fields and grasslands in Nova Scotia. Adults were collected from foliage of a variety of coniferous and deciduous tree species, herbaceous vegetation and rotting mushrooms. In New Brunswick, one adult was collected from a decaying mushroom in a conifer forest with white pine (*Pinus strobus* L.) and spruce, another in a Lindgren funnel trap deployed in an old red oak forest. Although this species appears to be common and widespread in the lower mainland of Nova Scotia (Majka 2010), it was found at only two localities (one specimen at each locality) in New Brunswick. Adults were captured during August in New Brunswick.

##### Distribution in Canada and Alaska.

MB, ON, QC, **NB**, NS (Bousquet 1991; Majka 2010).

### Family Tetratomidae Billberg, 1820

Members of the Tetratomidae (the polypore fungus beetles), as their name implies, feed on fruiting bodies of Polyporaceae and Tricholomataceae, and are commonly found under fungus grown bark or in softer shelf fungi (Young and Pollock 2002). Adults usually feed on the surface of the fungi, whereas the larvae bore into and feed on the tissues. Majka and Pollock (2006) reviewed the Tetratomidae and other saproxylic beetles (Melandryidae, Synchroidae, Scraptiidae) of the Maritime provinces, summarized the known bionomics, and discussed the fauna in the context of potential impact that forest management practices may have on members of these families in the region. Only one species, *Penthe pimelia* (Fabricius) was reported as occurring in New Brunswick by LeSage (1991a) and Majka and Pollock (2006). Later, Pollock (2008) reported *Pseudoholostrophus discolour* (Horn) from the province. Here, we report seven additional species of Tetratomidae from New Brunswick (Table 1.). Four of these species, *Tetratoma variegata* Casey, *Pisenus humeralis* Kirby, *Hallomenus serricornis* LeConte, and *Synstrophus repandus* (Horn), are newly recorded for the Maritime provinces.

### Subfamily Tetratominae Billberg, 1820

#### Tetratoma tessellata

Melsheimer, 1844

http://species-id.net/wiki/Tetratoma_tessellata

Map 8

##### Material examined.

**New Brunswick, Carleton Co.**, Jackson Falls, Bell Forest, 46.2200°N, 67.7231°W, 27.VI.5.VII.2008, 12–19.VII.2008, 19–28.VII.2008, 28.VII–6.VIII.2008, 6–14.VIII.2008, R. P. Webster, mature hardwood forest, Lindgren funnel traps (8, AFC, NBM, RWC); same locality and habitat data but 21–28.VI.2009, 7–14.VII.2009, 14–19.VII.2009, 19–31.VII.2009, 31.VII-7.VIII.2009, 7–12.VIII.2009, R. Webster & M.-A. Giguère, Lindgren funnel traps (12, AFC, RWC). **Charlotte Co.**, 10 km NW of New River Beach, 45.2110°N, 66.6170°W, 16–30.VI.2010, 16–26.VII.2010, R. Webster & C. MacKay, old growth eastern white cedar forest, Lindgren funnel traps (2, AFC). **Queens Co.**, Cranberry Lake P.N.A., 46.1125°N, 65.6075°W, 21–28.VII.2009, 6–14.VIII.2009, R. Webster & M.-A. Giguère, old red oak forest, Lindgren funnel traps (2, AFC, RWC); same locality data and forest type, 7–22.VI.2011, 20.VII-4.VIII.2011, M. Roy & V. Webster, Lindgren funnel traps (2, NBM); Grand Lake Meadows P.N.A., 45.8227°N, 66.1209°W, 5–19.VII.2011, 5–17.VIII.2011, M. Roy & V. Webster, old silver maple swamp and seasonally flooded marsh, Lindgren funnel traps (2, AFC, NBM). **Restigouche Co.**, Mount Carleton Provincial Park, 47.4042°N, 66.9189°W, 3.IX.2006, R. P. Webster, old hardwood forest, on slightly dried *Pleurotus* sp. on dead, standing sugar maple (1, RWC);Dionne Brook P.N.A., 47.9030°N, 68.3503°W, 30.V-15.VI.2011, 14–28.VII.2011, M. Roy & V. Webster, old-growth northern hardwood forest, Lindgren funnel traps (2, NBM); same locality and collectors but 47.9064°N, 68.3441°W, 27.VI-14.VII.2011, old-growth white spruce and balsam fir forest, Lindgren funnel trap (1, NBM). **Sunbury Co.**, Acadia Research Forest, 45.9866°N, 66.3841°W, 29.VII-4.VIII.2009, 4–11.VIII.2009, R. Webster & M.-A. Giguère, mature (110 year-old) red spruce forest with scattered red maple and balsam fir, Lindgren funnel traps (3, AFC). **York Co.**, 15 km W of Tracy off Rt. 645, 45.6848°N, 66.8821°W, 29.VII-4.VIII.2009, R. Webster & M.-A. Giguère, old red pine forest, Lindgren funnel trap (1, AFC); same locality data and forest type, 6–18.VII.2011, M. Roy & V. Webster, Lindgren funnel trap (1, NBM).

**Map 8.**
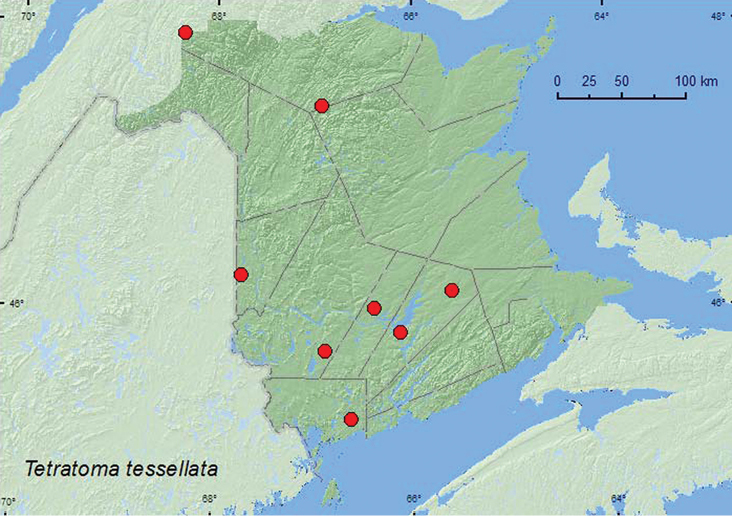
Collection localities in New Brunswick, Canada of *Tetratoma tessellata*.

##### Collection and habitat data.

*Tetratoma tessellata* was found in various forest types in New Brunswick. Adults were found in mature and old hardwood forests with American beech, yellow birch, and sugar maple, an old-growth northern hardwood forest with sugar maple and yellow birch, an old red oak forest, an old silver maple (*Acer saccharinum* L.) swamp, an old eastern white cedar (*Thuja occidentalis* L.) forest, a mature (110-year-old) red spruce stand, and an old (180-year-old) red pine forest. Most adults were captured in Lindgren funnel traps. One individual was collected from a slightly dried *Pleurotus* sp. on a dead, standing sugar maple. Most records reported from Nova Scotia by Majka and Pollock (2006) were caught with flight intercept traps in both coniferous and deciduous forests. Adults were collected during June, July, August, and September.

##### Distribution in Canada and Alaska.

ON, QC, **NB**, NS (LeSage 1991a; Majka and Pollock 2006).

#### Tetratoma variegata

Casey, 1900**

http://species-id.net/wiki/Tetratoma_variegata

Map 9

##### Material examined.

**New Brunswick, Restigouche, Co.**, Dionne Brook P.N.A., 47.9064°N, 68.3441°W, 31.V-15.VI.2011, 28.VII-4.VIII.2011, M. Roy & V. Webster, old-growth white spruce and balsam fir forest, Lindgren funnel traps (11, AFC, NBM, RWC).

**Map 9.**
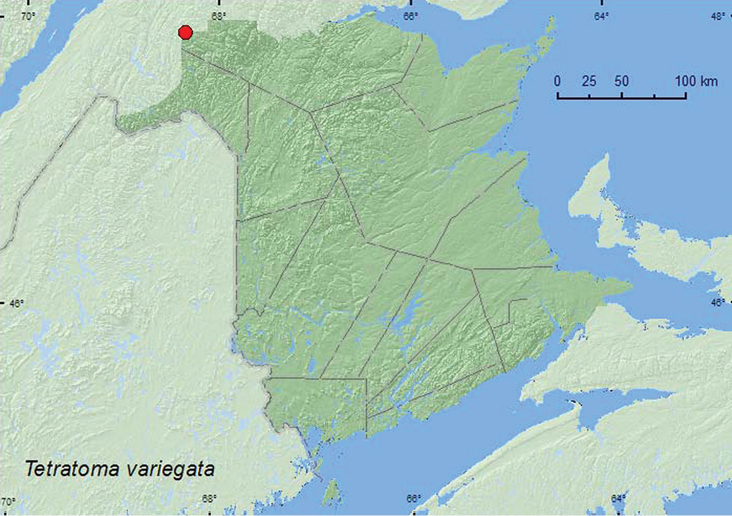
Collection localities in New Brunswick, Canada of *Tetratoma variegata*.

##### Collection and habitat data.

This species was captured in Lindgren funnel traps deployed in an old-growth white spruce and balsam fir forest. Adults were captured during June, July, and August (most during June).

##### Distribution in Canada and Alaska.

QC, **NB**, NF (LeSage 1991a).

### Subfamily Piseninae Miyatake, 1960

#### Pisenus humeralis

(Kirby, 1837)**

http://species-id.net/wiki/Pisenus_humeralis

Map 10

##### Material examined.

**New Brunswick, Carleton Co.**, Meduxnekeag Valley Nature Preserve, 46.1900°N, 67.6700°W, 7.VI.2007, R. P. Webster, mature hardwood forest, in large (from previous year) fleshy polypore fungus on beech log (11, NBM, RWC); Jackson Falls, Bell Forest, 46.2200°N, 67.7231°W, 23–28.IV.2009, R. Webster & M.-A. Giguère, mature hardwood forest, Lindgren funnel trap (1, AFC). **Queens Co.**, Cranberry Lake P.N.A, 46.1125°N, 65.6075°W, 14.VIII.2009, R. Webster & M.-A. Giguère, margin of old red oak forest, in bracket fungi on sun-exposed stump (1, AFC).

**Map 10.**
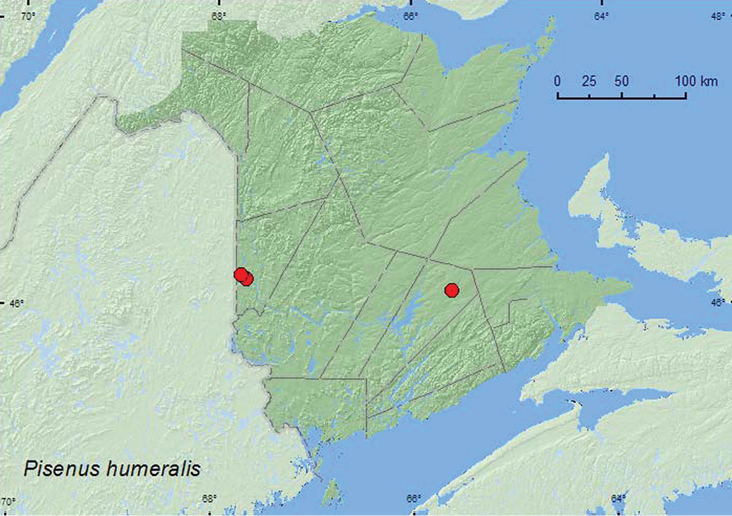
Collection localities in New Brunswick, Canada of *Pisenus humeralis*.

##### Collection and habitat data.

This species was found in mature hardwood forests with sugar maple and American beech, and in an old red oak forest. Eleven individuals (over 30 individuals observed) were collected from several large, decaying (from previous year), fleshy polypore fungi on an American beech log (tree had fallen during previous winter). One individual was collected from small bracket fungi on a sun-exposed stump, and one adult was captured in a Lindgren funnel trap. Adults were collected during April, June, and August.

##### Distribution in Canada and Alaska.

ON, QC, **NB** (LeSage 1991a).

### Subfamily Penthinae Lacordaire, 1859

#### Penthe obliquata

(Fabricius, 1801)

http://species-id.net/wiki/Penthe_obliquata

Map 11

##### Material examined.

**New Brunswick, Albert Co.**, Caledonia Gorge P.N.A., 45.8257°N, 64.7791°W, 6.VII.2011, R. P. Webster, old hardwood forest (sugar maple and beech), in *Polyporus varius* on dead standing sugar maple (1, NBM). **Carleton Co.**, Jackson Falls, Bell Forest, 46.2200°N, 67.7231°W, 13.VII.2004, K. Bredin, J. Edsall, & R. Webster, mature hardwood forest, u.v. light trap (1, RWC); same locality data and forest type, 16.IX.2006, R. P. Webster, on fleshy polypore fungi on standing dead beech tree (1, RWC); same locality, collector, and forest type, 4–12.VI.2009, 19–27.VI.2008, Lindgren funnel traps (2, AFC); same locality and forest type, 16–21.VI.2009, 21–28.VI.2009, 28.VI-7.VII.2009, R. Webster & M.-A. Giguère, Lindgren funnel traps (6, AFC); Meduxnekeag Valley Nature Preserve, 46.1931°N, 67.6825°W, 10.VI.2005, R. P. Webster, floodplain forest, u.v. light trap (1, NBM). **Charlotte Co.**, 10 km NW of New River Beach, 45.2110°N, 66.6170°W, 10–26.V.2010, R. Webster & C. MacKay, old growth eastern white cedar forest, Lindgren funnel trap (1, AFC). **Northumberland Co.**, 12 km SSE of Upper Napan near Goodfellow Brook, 46.8943°N, 65.3810°W, 23.V.2007, R. P. Webster, recent clear-cut, under bark of spruce log (1, RWC). **Queens Co.**, Grand Lake near Scotchtown, 45.8762°N, 66.1816°W, 19.IX.2006, R. P. Webster, oak and maple forest, in fleshy polypore fungi on dead red oak (1, RWC); Cranberry Lake P.N.A, 46.1125°N, 65.6075°W, 25.VI-1.VII.2009, 1–10.VII.2009, 10–15.VII.2009, 15–21.VII.2009, R. Webster & M.-A. Giguère, old red oak forest, Lindgren funnel traps (4, AFC). **Restigouche Co.**, Jacquet River Gorge P.N.A., 47.7764°N, 66.1279°W, 14.VIII.2010, J. Goltz, mixed forest, in woody polypore on dead (standing) balsam fir (4, NBM); Dionne Brook P.N.A., 47.9030°N, 68.3503°W, 27.VI-14.VII.2011, M. Roy & V. Webster, old-growth northern hardwood forest, Lindgren funnel trap (1, NBM). **Sunbury Co.**, Lakeville Corner, 45.9007°N, 66.2423°W, 27.VIII.2006, R. P. Webster, silver maple swamp, among polypore fungi on poplar log (1, RWC); Acadia Research Forest, 45.9799°N, 66.3394°W, 18.VI.2007, R. P. Webster, Rd. 7 control, mature red spruce and red maple forest, in fleshy polypore fungi on stump (1, NBM); same locality but 45.9866°N, 66.3841°W, 24–30.VI.2009, 30.VI-8.VII.2009, 8–13.VII.2009, R. Webster & M.-A. Giguère, mature (110-year-old) red spruce forest with scattered red maple and balsam fir, Lindgren funnel traps (5, AFC). **York Co.**, 15 km W of Tracy off Rt. 645, 45.6848°N, 66.8821°W, 21–28.VI.2009, 28.VI-7.VII.2009, R. Webster & M.-A. Giguère, old red pine forest, Lindgren funnel traps (2, AFC); same locality data and forest type, 8–20.VI.2011, M. Roy & V. Webster, Lindgren funnel trap (1, NBM); 14 km WSW of Tracy, S of Rt. 645, 45.6741°N, 66.8661°W, 30.VI-13.VII.2010, R. Webster & C. MacKay, old mixed forest with red and white spruce, red and white pine, balsam fir, eastern white cedar, red maple, and *Populus* sp., Lindgren funnel trap (1, AFC).

**Map 11.**
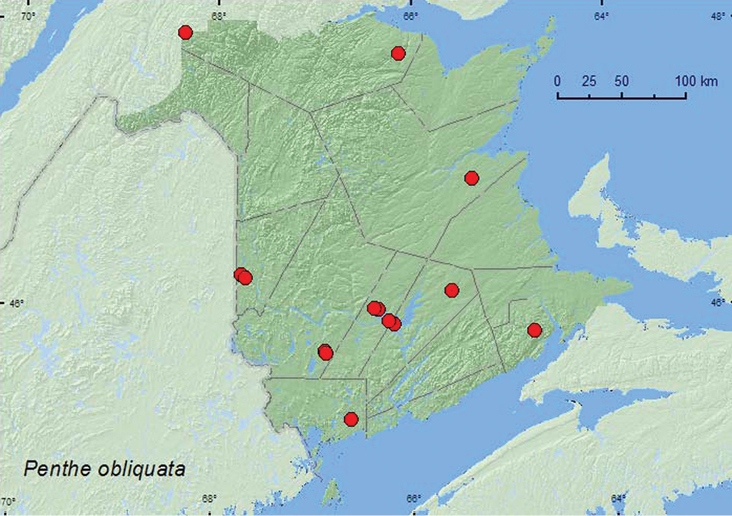
Collection localities in New Brunswick, Canada of *Penthe obliquata*.

##### Collection and habitat data.

*Penthe obliquata* was found in mature hardwood forests with American beech and sugar maple, an old-growth northern hardwood forest, a floodplain forest with black ash (*Fraxinus nigra* Marsh.), butternut, and red maple, an old red oak forest, a red oak and red maple stand, a silver maple swamp, a mature (110-year-old) red spruce stand, an old (180-year-old) red pine forest, an old eastern white cedar forest, and in mixed forests. Many adults were captured in Lindgren funnel traps deployed in the above forest types. Adults with specific collection data were collected from polypore fungi (bracket fungi) on standing dead American beech trees and poplar logs, in fleshy polypore fungi on a dead, standing red oak and on a stump, from a woody polypore on a dead, standing balsam fir, from *Polyporus varius* Fr. on a dead, standing sugar maple, and from under bark of a spruce log. Majka and Pollock (2006) reported this species from under bark of a variety of conifer species in Nova Scotia. They also reported adults from belted polypore, *Fomitopsis pinicola* (Fr.) Kar., on conifers. Adults from New Brunswick were captured during May, June, July, August, and September,

##### Distribution in Canada and Alaska.

ON, QC, **NB**, NS (LeSage 1991a; Majka and Pollock 2006).

### Subfamily Hallomeninae Gistel, 1848

#### Hallomenus serricornis

LeConte, 1878**

http://species-id.net/wiki/Hallomenus_serricornis

Map 12

##### Material examined.

**New Brunswick, Sunbury Co.**, Acadia Research Forest, 45.9866°N, 66.3841°W, 8–13.VII.2009, R. Webster & M.-A. Giguère, mature (110 year-old) red spruce forest with scattered red maple and balsam fir, Lindgren funnel trap (1, RWC). **York Co.**, Charters Settlement, 45.8395°N, 66.7391°W, 1.VIII.2004, R. P. Webster, mixed forest, u.v. light (1, RWC); 15 km W of Tracy off Rt. 645, 45.6848°N, 66.8821°W, 4–16.VI.2010, R. Webster & C. MacKay, old red pine forest, Lindgren funnel trap (1, RWC).

**Map 12.**
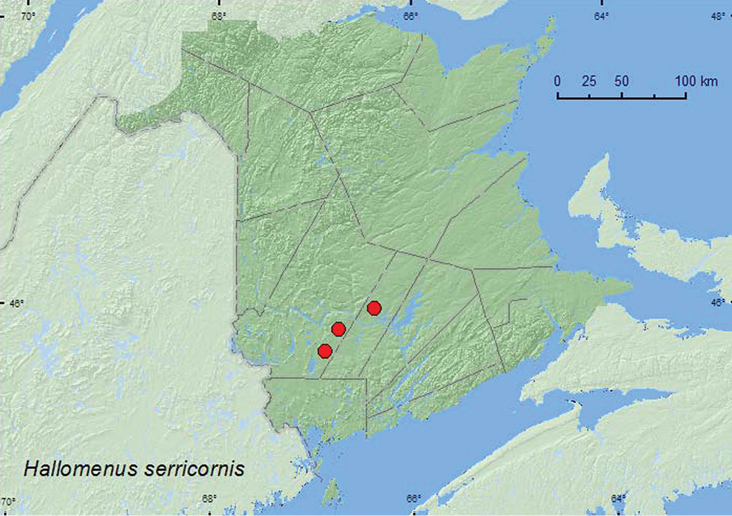
Collection localities in New Brunswick, Canada of *Hallomenus serricornis*.

##### Collection and habitat data.

This species was captured in Lindgren funnel traps deployed in a mature red spruce forest and an old red pine forest. One individual was collected at and ultraviolet light in a mixed forest. Adults were collected during June, July, and August.

##### Distribution in Canada and Alaska.

BC, AB, ON, QC, **NB** (LeSage 1991b).

### Subfamily Eustrophinae Gistel, 1848

**Tribe Eustrophini Gistel, 1848**

#### Eustrophus tomentosus

Say, 1826

http://species-id.net/wiki/Eustrophus_tomentosus

Map 13

##### Material examined.

**New Brunswick, Carleton Co.**, Jackson Falls, Bell Forest, 46.2200°N, 67.7231°W, 4–12.VI.2008, 5–12.VII.2008, R. P. Webster, mature hardwood forest, Lindgren funnel traps (2, RWC). **Queens Co.**, Grand Lake near Scotchtown, 45.8762°N, 66.1816°W, 3.VI.2007, R. P. Webster, oak and maple forest, under bark of dead red oak (1, RWC); Grand Lake Meadows P.N.A., 45.8227°N, 66.1209°W, 26.VII-7.VIII.2010, R. Webster & C. MacKay, old silver maple forest with green ash and seasonally flooded marsh, Lindgren funnel trap (1, RWC). **York Co.**, Canterbury, near Browns Mountain Fen, 45.8876°N, 67.6560°W, 3.VIII.2006, R. P. Webster, mature hardwood forest, on partially dried *Pleurotus* species on dead standing sugar maple (1, RWC).

**Map 13.**
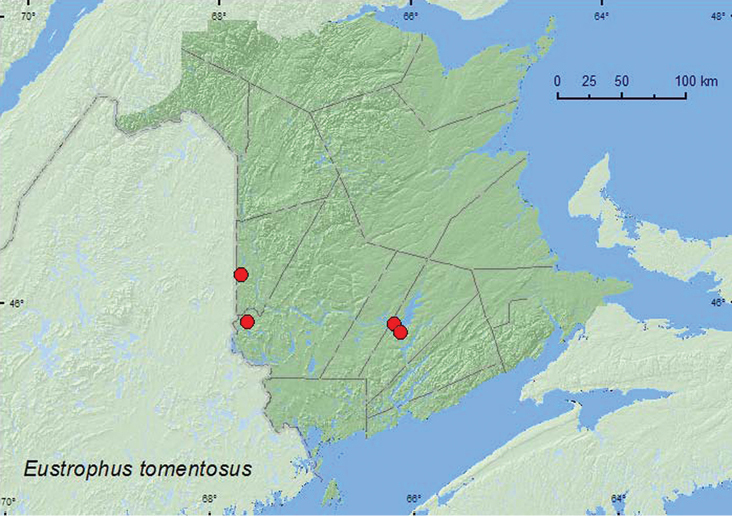
Collection localities in New Brunswick, Canada of *Eustrophus tomentosus*.

##### Collection and habitat data.

This species was found in hardwood forests in New Brunswick. These included a mature hardwood forests with American beech and sugar maple, a red oak and red maple forest, and a silver maple forest/swamp. Adults were found under bark of red oak, and in a partially dried *Pleurotus* mushroom on a dead, standing sugar maple, A few adults were captured in Lindgren funnel traps. This species has been reported from under bark of dead trees and is attracted to sap (Chantal 1985). Adults were collected during June, July, and August.

##### Distribution in Canada and Alaska.

BC, ON, QC, **NB**, NS (LeSage 1991b; Majka and Pollock 2006).

#### Synstrophus repandus

(Horn, 1888)**

http://species-id.net/wiki/Synstrophus_repandus

Map 14

##### Material examined.

**New Brunswick, Carleton Co.**, Meduxnekeag Valley Nature Preserve, 46.1887°N, 67.6735°W, 13.VI.2010, 18.VI.2010, R. P. Webster, hardwood forest, in *Laetiporus sulphureus* (3, NBM, RWC). **York Co.**, Canterbury, 45.8841°N, 67.6428°W, 8.VI.2004, D. Sabine & R. Webster, mature hardwood forest, sweeping foliage along forest trail (1, RWC); Canterbury, near Browns Mountain Fen, 45.8876°N, 67.6560°W, 3.VIII.2006, R. Webster & M.-A. Giguère, mature hardwood forest, on partially dried *Pleurotus* species on dead standing sugar maple (2, NBM, RWC); Charters Settlement, 45.8340°N, 66.7450°W, 17.VIII.2008, R. P. Webster, mature mixed forest, on polypore fungi on dead standing *Populus* sp. (1, RWC); 15 km W of Tracy off Rt. 645, 45.6848°N, 66.8821°W, 4.V.2009, R. Webster & M.-A. Giguère, old red pine forest, under bark of red maple (1, RWC).

**Map 14.**
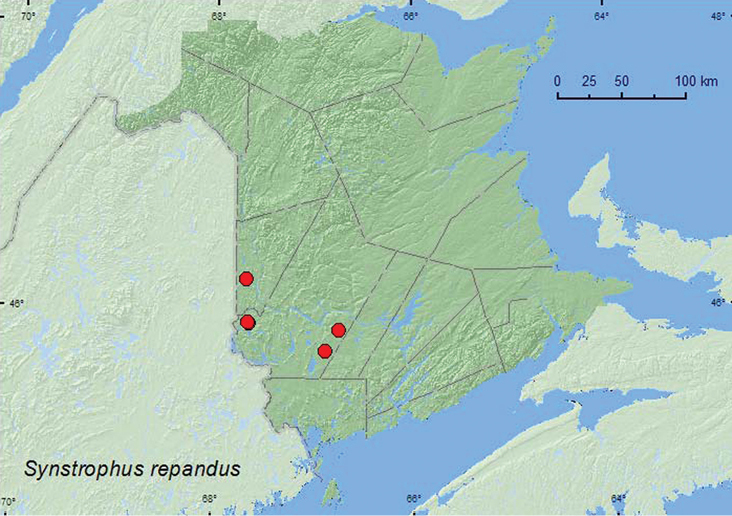
Collection localities in New Brunswick, Canada of *Synstrophus repandus*.

##### Collection and habitat data.

In New Brunswick, *Synstrophus repandus* was found in mature hardwood forests with sugar maple and American beech, a mature mixed forest, and in an old red pine forest. Adults were found in *Laetiporus sulphureus* (Fr.) Murr., partially dried *Pleurotus* mushrooms on a dead, standing sugar maple, in a polypore fungi on a dead, standing *Populus* sp., and under bark of red maple. One individual was swept from vegetation along a forest trail. Adults were collected during May, June, and August.

##### Distribution in Canada and Alaska.

BC, MB, ON, QC, **NB** (LeSage 1991b).

### Family Melandryidae Leach, 1815

The Melandryidae (the false darkling beetles) are either xylophagous (Serropalpini and Melandryini) or fungivores (Orchesiini), although fungi may be a significant portion of the diet of the xylophagous species (Pollock 2002). Majka and Pollock (2006) reviewed the Melandryidae of the Maritime provinces, summarized the known bionomics, and discussed the fauna in the context of potential impact of forest management practices in the region. They reported 16 species from New Brunswick; *Enchodes sericea* (Haldeman), *Prothalpia undata* LeConte, and *Emmesia connectens* Newman were reported as new to the province. Here, we report 10 additional species for the province. *Microscapha clavicornis* LeConte and *Zilora nuda* Provancher are newly recorded for the Maritime provinces (Table 1).

### Subfamily Melandryinae Leach, 1815

**Tribe Hypulini Gistel, 1848**

#### Symphora flavicollis

(Haldeman, 1848)

http://species-id.net/wiki/Symphora_flavicollis

Map 15

##### Material examined.

**New Brunswick, Carleton Co.**,Jackson Falls,Bell Forest, 46.2200°N, 67.7231°W, 27.VI-6.VII.2008, 6–12.VII.2009, R. P. Webster, mature hardwood forest, Lindgren funnel traps (2, AFC); same locality and forest type, 28.VI–7.VII.2009, R. Webster & M.-A. Giguère, Lindgren funnel trap (1, RWC). **Queens Co.**, Cranberry Lake P.N.A, 46.1125°N, 65.6075°W, 29.VI–7.VII.2011, 7–13.VII.2011, M. Roy & V. Webster, old red oak forest, Lindgren funnel traps (2, RWC). **Restigouche Co.**, Dionne Brook P.N.A., 47.9030°N, 68.3503°W, 27.VI-14.VII.2011, M. Roy & V. Webster, old-growth northern hardwood forest, Lindgren funnel trap (1, NBM). **Sunbury Co.**, Burton near Sunpoke Lake, 45.7658°N, 66.5546°W, 27.VII.2007, R. P. Webster, red oak and red maple forest, u.v. light (2, RWC); Acadia Research Forest, 45.9866°N, 66.3841°W, 30.VI-8.VII.2009, R. Webster & M.-A. Giguère, mature (110-year-old) red spruce forest with scattered red maple and balsam fir, Lindgren funnel trap (1, RWC). **York Co.**, Charters Settlement, 45.8430°N, 66.7275°W, 12.VII.2005, R. P. Webster, regenerating mixed forest, beating foliage (1, RWC); Canterbury, near Browns Mountain Fen, 45.8978°N, 67.6273°W, 3.VII.2005, R. Webster & M.-A. Giguère, mixed forest, beating foliage (1, RWC); 15 km W of Tracy off Rt. 645, 45.6848°N, 66.8821°W, 4–16.VI.2010, R. Webster & C. MacKay, old red pine forest, Lindgren funnel trap (1, RWC).

**Map 15.**
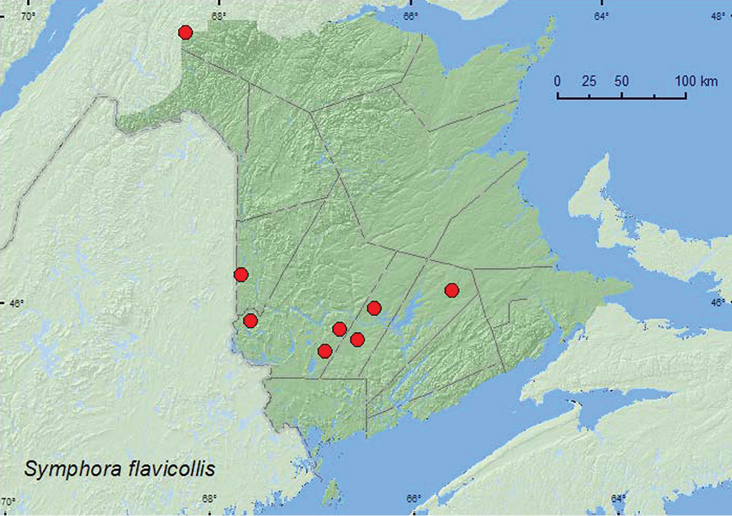
Collection localities in New Brunswick, Canada of *Symphora flavicollis*.

##### Collection and habitat data.

*Symphora flavicollis* was found in a mature hardwood forest with sugar maple and American beech, a red oak and red maple stand, an old red oak forest, an old-growth northern hardwood forest, a regenerating (20-year-old) mixed forest, mixed forests, a mature red spruce forest, and an old red pine forest. Adults were captured in Lindgren funnel traps, at ultraviolet light, and by beating foliage. Majka and Pollock (2006) reported this species from various coniferous and hardwood forest types in Nova Scotia. This species was collected during June and July.

##### Distribution in Canada and Alaska.

MB, ON, QC, **NB**, PE, NS (LeSage 1991b; Majka and Pollock 2006).

#### Symphora rugosa

(Haldeman, 1848)

http://species-id.net/wiki/Symphora_rugosa

Map 16

##### Material examined.

**New Brunswick, Carleton Co.**, Meduxnekeag Valley Nature Preserve, 46.1931°N, 67.6825°W, 25.VI.2007, 5.VII.2008, R. P. Webster, floodplain forest, sweeping foliage (6, RWC). **Charlotte Co.**, 10 km NW of New River Beach, 45.2110°N, 66.6170°W, 29.VI–16.VII.2009, R. Webster & C. MacKay, old growth eastern white cedar forest, Lindgren funnel trap (1, AFC). **Queens Co.**, Grand Lake Meadows P.N.A., 45.8227°N, 66.1209°W, 19.VII-5.VIII.2011, M. Roy & V. Webster, old silver maple forest with green ash and seasonally flooded marsh, Lindgren funnel trap (1, RWC). **Restigouche Co.**, Dionne Brook P.N.A., 47.9030°N, 68.3503°W, 14–28.VII.2011, M. Roy & V. Webster, old-growth northern hardwood forest, Lindgren funnel trap (1, RWC). **Saint John Co.**, Dipper Harbour, 45.1154°N, 66.3725°W, 6.VII.2008, R. P. Webster, red spruce forest on outcrop, on red spruce foliage (beating foliage) (1, RWC). **York Co.**, 15 km W of Tracy off Rt. 645, 45.6848°N, 66.8821°W, 30.VI-13.VII.2010, R. Webster & K. Burgess, old red pine forest, Lindgren funnel trap (1, AFC).

**Map 16.**
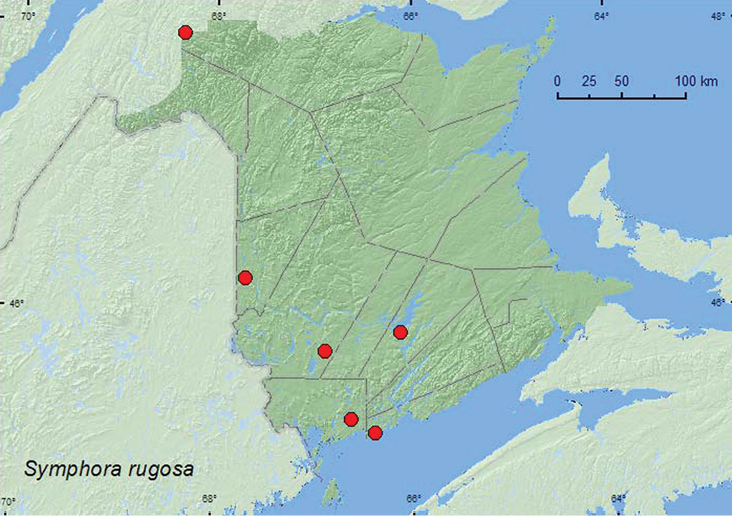
Collection localities in New Brunswick, Canada of *Symphora rugosa*.

##### Collection and habitat data.

This species was found in a floodplain forest with black ash and butternut, an old silver maple swamp, an old-growth northern hardwood forest, an old-growth eastern white cedar forest/swamp, a red spruce stand on a rock outcrop, and an old red pine forest. Adults were collected by sweeping foliage, beating red spruce foliage, and in Lindgren funnel traps. This species was collected during June, July, and August.

##### Distribution in Canada and Alaska.

ON, QC, **NB**, NS (LeSage 1991b).

### Tribe Orchesiini Mulsant, 1856

#### Microscapha clavicornis

LeConte, 1866**

http://species-id.net/wiki/Microscapha_clavicornis

Map 17

##### Material examined.

**New Brunswick, York Co.**, 15 km W of Tracy off Rt. 645, 45.6848°N, 66.8821°W, 29.VII-4.VIII.2009, R. Webster & M.-A. Giguère, old red pine forest, Lindgren funnel traps (2, AFC, RWC).

**Map 17.**
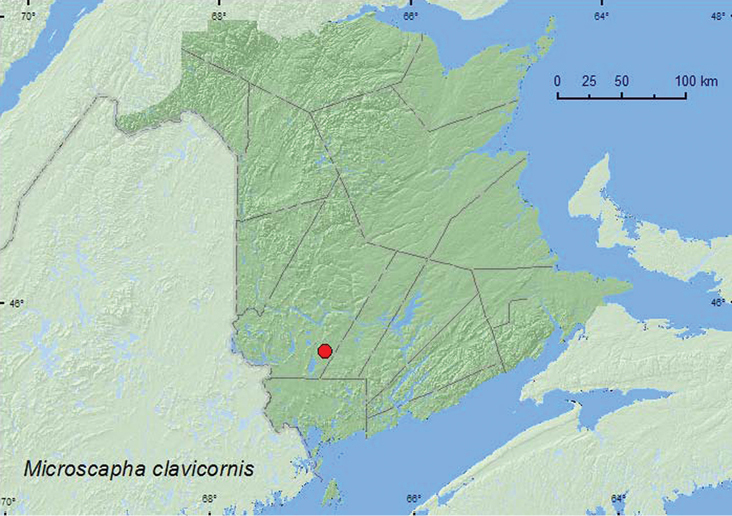
Collection localities in New Brunswick, Canada of *Microscapha clavicornis*.

##### Collection and habitat data.

Two specimens of this rare species were captured between late July and early August in Lindgren funnel traps deployed in an old red pine forest.

##### Distribution in Canada and Alaska.

QC, **NB** (LeSage 1991b).

#### Orchesia cultriformis

Laliberté, 1967

http://species-id.net/wiki/Orchesia_cultriformis

Map 18

##### Material examined.

**New Brunswick, Carleton Co.**, Meduxnekeag Valley Nature Preserve, 46.1910°N, 67.6740°W, 31.VIII.2006, R. P. Webster, mature mixed forest, in polypore fungi (1, RWC); Jackson Falls, Bell Forest, 46.2200°N, 67.7231°W, 12–19.VII.2008, 6–14.VIII.2008, R. P. Webster, mature hardwood forest, Lindgren funnel traps (2, AFC); same locality and habitat data but 21–28.VI.2009, 7–14.VII.2009, 19–31.VII.2009, R. Webster & M.-A. Giguère, Lindgren funnel traps (3, AFC). **Charlotte Co.**, 10 km NW of New River Beach, 45.2110°N, 66.6170°W, 29.VI-16.VII.2010, R. Webster & C. MacKay, old growth eastern white cedar forest, Lindgren funnel trap (1, AFC). **Queens Co.**, Cranberry Lake P.N.A., 46.1125°N, 65.6075°W, 18–25.VI.2009, 25.VI-1.VII.2009, 15–21.VII.2009, 28.VII-6.VIII.2009, 6–14.VIII.2009, R. Webster & M.-A. Giguère, old red oak forest, Lindgren funnel traps (9, AFC); Grand Lake Meadows P.N.A., 45.8227°N, 66.1209°W, 5–17.VIII.2011, M. Roy & V. Webster, old silver maple forest with green ash and seasonally flooded marsh, Lindgren funnel trap (1, NBM). **Sunbury Co.**, Acadia Research Forest, 45.9866°N, 66.3841°W, 21–29.VII.2009, 29.VII–4.VIII.2009, R. Webster & M.-A. Giguère, mature (110-year-old) red spruce forest with scattered red maple and balsam fir, Lindgren funnel traps (3, AFC). **York Co.**, Charters Settlement, 45.8395°N, 66.7391°W, 4.VII.2005, R. P. Webster, mixed forest, u.v. light; same locality and collector but 45.8286°N, 66.7365°W, 25.VII.2006, 6.VIII.2006, mature mixed forest, on polypore fungi on dead standing beech and dead standing hemlock (4, RWC); 15 km W of Tracy off Rt. 645, 45.6848°N, 66.8821°W, 20–29.VII.2009, 4–11.VIII.2009, R. Webster & M.-A. Giguère, old red pine forest, Lindgren funnel traps (2, AFC); 14 km WSW of Tracy, S of Rt. 645, 45.6741°N, 66.8661°W, 30.VI-13.VII.2010 R. Webster & C. MacKay, old mixed forest with red and white spruce, red and white pine, balsam fir, eastern white cedar, red maple, and *Populus* sp., Lindgren funnel trap (1, AFC).

**Map 18.**
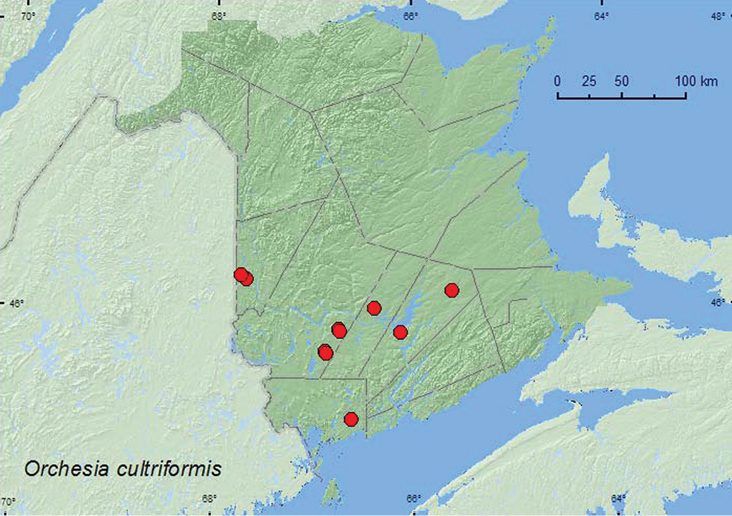
Collection localities in New Brunswick, Canada of *Orchesia cultriformis*.

##### Collection and habitat data.

This species was found in various forest types in New Brunswick. These included hardwood forests with sugar maple and American beech, an old red oak stand, mixed forests, an old eastern white cedar forest/swamp, a red spruce forest, and an old red pine forest. Most adults were captured in Lindgren funnel traps. Adults with specific habitat data were collected from polypore fungi and from polypore fungi on a dead, standing American beech and a dead, standing eastern hemlock (*Tsuga canadensis* (L.) Carr.). One individual was collected at an ultraviolet light. This species and *Orchesia castanea* (Melsheimer) have an amazing jumping ability, and adults often jumped out of a 15 cm high sifting box, resulting in the loss of many specimens. Adults were collected during June, July, and August.

##### Distribution in Canada and Alaska.

NT, SK, MB, QC, **NB**, NS (LeSage 1991b).

#### Orchesia ovata

Laliberté, 1967

http://species-id.net/wiki/Orchesia_ovata

Map 19

##### Material examined.

**New Brunswick, Carleton Co.**, Jackson Falls, Bell Forest, 46.2200°N, 67.7231°W, 28.VI–7.VII.2009, R. Webster & M.-A. Giguère, mature hardwood forest, Lindgren funnel trap (1, RWC). **Queens Co.**, Cranberry Lake P.N.A, 46.1125°N, 65.6075°W, 1–10.VII.2009, 10–15.VII.2009, 15–21.VII.2009, 21–28.VII.2009, R. Webster & M.-A. Giguère, old red oak forest, Lindgren funnel traps (5, RWC); Grand Lake Meadows P.N.A., 45.8227°N, 66.1209°W, 5–19.VII.2011, 5–17.VIII.2011, 17–30.VIII.2011, M. Roy & V. Webster, old silver maple forest and seasonally flooded marsh, Lindgren funnel traps (3, AFC, NBM). **Restigouche Co.**, Dionne Brook P.N.A., 47.9030°N, 68.3503°W, 14–28.VII.2011, M. Roy & V. Webster, old-growth northern hardwood forest, Lindgren funnel traps (2, AFC, NBM); same locality and collectors but 47.9064°N, 68.3441°W, 14–28.VII.2011, old-growth white spruce and balsam fir forest, Lindgren funnel traps (1, NBM). **Sunbury Co.** Burton, Sunpoke Lake, 45.7658°N, 66.5546°W, 26.VII-1.VIII.2008, R. P. Webster, oak forest with scattered white pine, Lindgren funnel trap (1, RWC); *ca*. 2.5 km S of Beaver Dam, 45.7735°N, 66.6852°W, 13.VIII.2008, R. P. Webster, powerline-right-of-way, sweeping foliage (1, RWC). **York Co.**, Canterbury, 45.8972°N, 67.6272°W, 21.VII.2004, D. Sabine, J. Edsall, K. Bredin, & R. Webster, mixed forest with cedar, sweeping foliage near small stream (1, RWC).

**Map 19.**
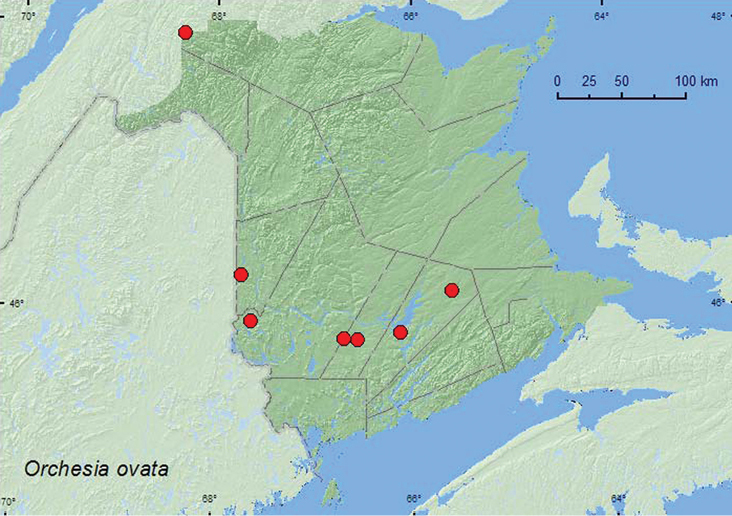
Collection localities in New Brunswick, Canada of *Orchesia ovata*.

##### Collection and habitat data.

In New Brunswick, *Orchesia ovata* was found in a mature hardwood forest with sugar maple and American beech, an old-growth northern hardwood forest with sugar maple and yellow birch, an old red oak forest, an oak forest with scattered white pine (*Pinus strobus* L.), an old-growth white spruce and balsam fir forest, a mixed forest, and along a powerline right-of-way. Most adults were captured in Lindgren funnel traps. A few individuals were swept from foliage. Elsewhere, this species has been found on wood covered with decaying foliage of red maple (Laliberté 1966). Adults were collected during June, July, and August.

##### Distribution in Canada and Alaska.

ON, QC, **NB**, NS (LeSage 1991b; Majka and Pollock 2006).*Orchesia ovata* was not listed by LeSage (1991a) as occurring in New Brunswick. Majka and Pollock (2006) reported this species from New Brunswick in Table 1 but did not include any data to support the record. The above records establish its presence in the province.

### Tribe Serropalpini Latreille, 1829

#### Enchodes sericea

(Haldeman, 1848)

http://species-id.net/wiki/Enchodes_sericea

Map 20

##### Material examined.

**Additional New Brunswick records. Carleton Co.**,Jackson Falls, Bell Forest, 46.2200°N, 67.7231°W, 19–27.VI.2008, 5–12.VII.2008, 12–19.VII.2008, 19–28.VII.2008, 6–14.VIII.2008, R. P. Webster, mature hardwood forest, Lindgren funnel traps (11, AFC, RWC); same locality and habitat data but 28.VI-7.VII.2009, 19–31.VII.2009, R. Webster & M.-A. Giguère, Lindgren funnel traps (4, AFC, RWC). **Queens Co.**, Cranberry Lake P.N.A, 46.1125°N, 65.6075°W, 25.VI-1.VII.2009, 1–10.VII.2009, 10–15.VII.2009, 15–21.VII.2009, 21–28.VII.2009, 28.VII-6.VIII.2009, 6–14.VIII.2009, R. Webster & M.-A. Giguère, old red oak forest, Lindgren funnel traps (13, AFC, RWC); Grand Lake Meadows P.N.A., 45.8227°N, 66.1209°W, 15–29.VI.2010, 29.VI–12.VII.2010, 12–26.VII.2010, R. Webster, C. MacKay, M. Laity, & R. Johns, old silver maple forest with green ash and seasonally flooded marsh, Lindgren funnel traps (7, AFC); same locality data and forest type, 5–19.VII.2011, 19.VII-5.VIII.2011, M. Roy & V. Webster, Lindgren funnel traps (4, AFC, NBM). **Restigouche Co.**, Dionne Brook P.N.A., 47.9030°N, 68.3503°W, 28.VII-9.VIII.2011, M. Roy & V. Webster, old-growth northern hardwood forest, Lindgren funnel trap (1, NBM). **York Co.**, 14 km WSW of Tracy, S of Rt. 645, 45.6741°N, 66.8661°W, 27.VII.2010 R. Webster & C. MacKay, old mixed forest with red and white spruce, red and white pine, balsam fir, eastern white cedar, red maple, and *Populus* sp., Lindgren funnel trap (1, AFC).

**Map 20.**
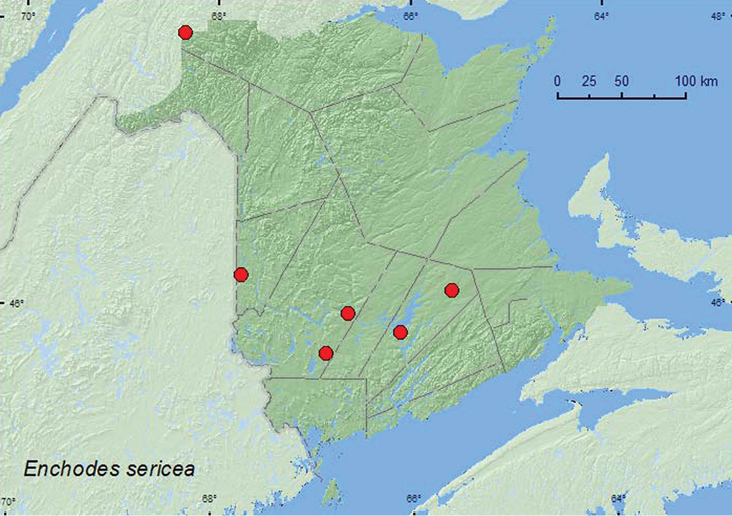
Collection localities in New Brunswick, Canada of *Enchodes sericea*.

##### Collection and habitat data.

In New Brunswick, most adults of *Enchodes sericea* were found in hardwood forests. These included a mature hardwood forest with sugar maple and American beech, an old red oak forest, an old silver maple forest/swamp, an old-growth northern hardwood forest, and an old mixed forest. All specimens were captured in Lindgren funnel traps. Adults were collected during June, July, and August.

##### Distribution in Canada and Alaska.

BC, AB, SK, MB, ON, QC, **NB**, NS (LeSage 1991b; Majka and Pollock 2006). This species was first reported from New Brunswick by Majka and Pollock (2006) based on a specimen collected in Fredericton by A.B. Baird in 1915. The above records are the first recent records of this species from New Brunswick and indicate this species is relatively common (41 specimens) in hardwood forests in the province.

#### Scotochroides antennatus

Mank, 1939

http://species-id.net/wiki/Scotochroides_antennatus

Map 21

##### Material examined.

**New Brunswick,** **Carleton Co.**, Jackson Falls, Bell Forest, 46.2200°N, 67.7231°W, 19–29.VII.2008, R. P. Webster, mature hardwood forest, Lindgren funnel trap (1, RWC). **Charlotte Co.**, 10 km NW of New River Beach, 45.2110°N, 66.6170°W, 16–26.VII.2010, R. Webster & C. MacKay, old growth eastern white cedar forest, Lindgren funnel traps (2, RWC).

**Map 21.**
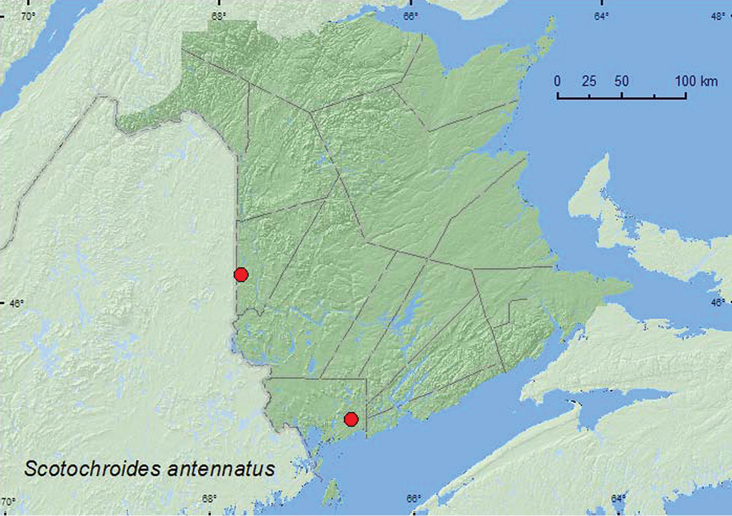
Collection localities in New Brunswick, Canada of *Scotochroides antennatus*.

##### Collection and habitat data.

Adults were captured in Lindgren funnel traps deployed in a mature hardwood forest with sugar maple and American beech, and in an old eastern white cedar forest/swamp. In Nova Scotia, this species was collected from conifer stands, mostly from flight intercept traps or by sweeping foliage (Majka and Pollock 2006). Adults were captured during July.

##### Distribution in Canada and Alaska.

QC, **NB**, NS (LeSage 1991b; Majka and Pollock 2006).

#### Phloeotrya fusca

(LeConte, 1878)

http://species-id.net/wiki/Phloeotrya_fusca

Map 22

##### Material examined.

**New Brunswick, Queens Co.**, Cranberry Lake P.N.A, 46.1125°N, 65.6075°W, 15–21.VII.2009, R. Webster & M.-A. Giguère, old red oak forest, Lindgren funnel trap (1, RWC). **Sunbury Co.**, Acadia Research Forest, 45.9866°N, 66.3841°W, 29.VII–4.VIII.2009, R. Webster & M.-A. Giguère, mature (110-year-old) red spruce forest with scattered red maple and balsam fir, Lindgren funnel trap (1, AFC). **York Co.**, 15 km W of Tracy off Rt. 645, 45.6848°N, 66.8821°W, 7–14.VII.2009, 4–11.VIII.2009, R. Webster & M.-A. Giguère, old red pine forest, Lindgren funnel traps (3, RWC); same locality and habitat data, 30.VI-13.VII.2010, R. Webster & K. Burgess, Lindgren funnel traps (6, AFC, RWC); 14 km WSW of Tracy, S of Rt. 645, 45.6741°N, 66.8661°W, 30.VI-13.VII.2010, 13–27.VII.2010, R. Webster, C. MacKay, & K. Burgess, old mixed forest with red and white spruce, red and white pine, balsam fir, eastern white cedar, red maple, and *Populus* sp., Lindgren funnel traps (3, AFC, RWC).

**Map 22.**
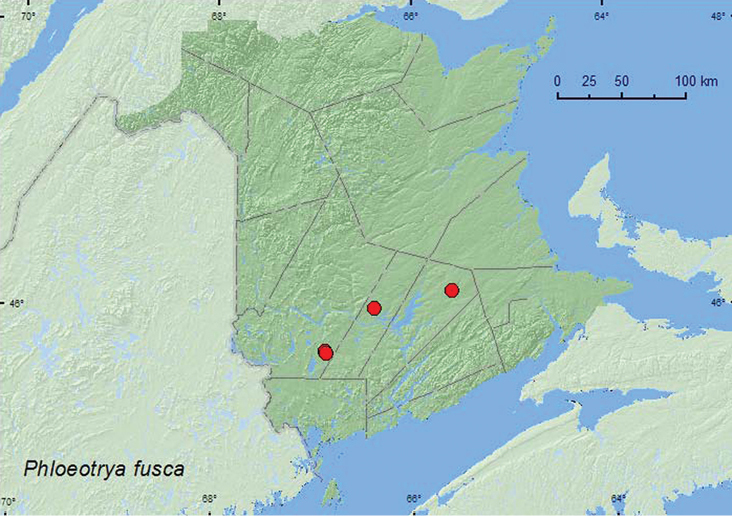
Collection localities in New Brunswick, Canada of *Phloeotrya fusca*.

##### Collection and habitat data.

Adults of this species were captured in Lindgren funnel traps deployed in an old red oak stand, an old (180-year-old) red pine stand, a mature (110-year-old) red spruce forest, and an old mixed forest. Majka and Pollock (2006) reported that this species was associated with balsam fir, red spruce, and white pine. In New Brunswick, *Phloeotrya fusca* was collected during July and August.

##### Distribution in Canada and Alaska.

QC, **NB**, PE, NS (LeSage 1991b; Majka and Pollock 2006).

#### Spilotus quadripustulatus

(Melsheimer, 1846)

http://species-id.net/wiki/Spilotus_quadripustulatus

Map 23

##### Material examined.

**New Brunswick, Carleton Co.**, Jackson Falls, Bell Forest, 46.2200°N, 67.7231°W, 4–7-14.VII.2009, 14–19.VII.2009, R. Webster & M.-A. Giguère, mature hardwood forest, Lindgren funnel traps (2, RWC). **Queens Co.**, Grand Lake Meadows P.N.A., 45.8227°N, 66.1209°W, 15–29.VI.2010, 29.VI-12.VII.2010, R. Webster, C. MacKay, M. Laity, & R. Johns, old silver maple forest with green ash and seasonally flooded marsh, Lindgren funnel traps (10, AFC, RWC); same locality data and forest type, 21.VI-5.VII.2011, 5–10.VII.2011, M. Roy & V. Webster, Lindgren funnel traps (5, NBM, RWC).

**Map 23.**
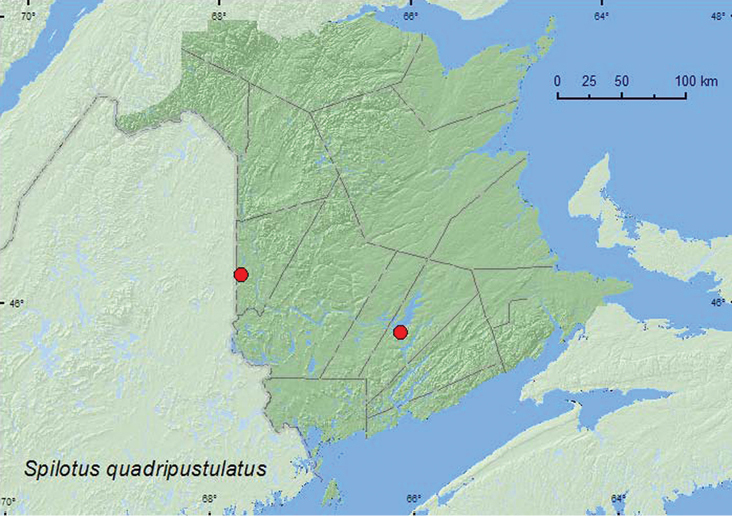
Collection localities in New Brunswick, Canada of *Spilotus quadripustulatus*.

##### Collection and habitat data.

Adults of *Spilotus quadripustulatus* were captured in Lindgren funnel traps deployed in a mature hardwood forest with sugar maple and American beech (2), and in an old silver maple forest/swamp (13). Adults were captured during June and July.

##### Distribution in Canada and Alaska.

QC, **NB**, NS (LeSage 1991b; Majka and Pollock 2006).

### Tribe Zilorini Desbrochers des Loges, 1900

#### Zilora hispida

LeConte, 1866

http://species-id.net/wiki/Zilora_hispida

Map 24

##### Material examined.

**New Brunswick, Queens Co.**, Cranberry Lake P.N.A, 46.1125°N, 65.6075°W, 13–25.V.2011, M. Roy & V. Webster, old red oak forest, Lindgren funnel trap (1, RWC). **Restigouche, Co.**, Dionne Brook P.N.A., 47.9064°N, 68.3441°W, 28.VII-9.VIII.2011, M. Roy & V. Webster, old-growth white spruce and balsam fir forest, Lindgren funnel trap (1, RWC). **York Co.**, Charters Settlement, 45.8286°N, 66.7365°W, 2.VI.2007, mature red spruce and red maple forest, under scolytid infested bark of red spruce (1, RWC); 14 km WSW of Tracy, S of Rt. 645, 45.6741°N, 66.8661°W, 10–26.V.2010, 2–16.VI.2010, R. Webster, C. MacKay, & K. Burgess, old mixed forest with red and white spruce, red and white pine, balsam fir, eastern white cedar, red maple, and *Populus* sp., Lindgren funnel traps (2, RWC).

**Map 24.**
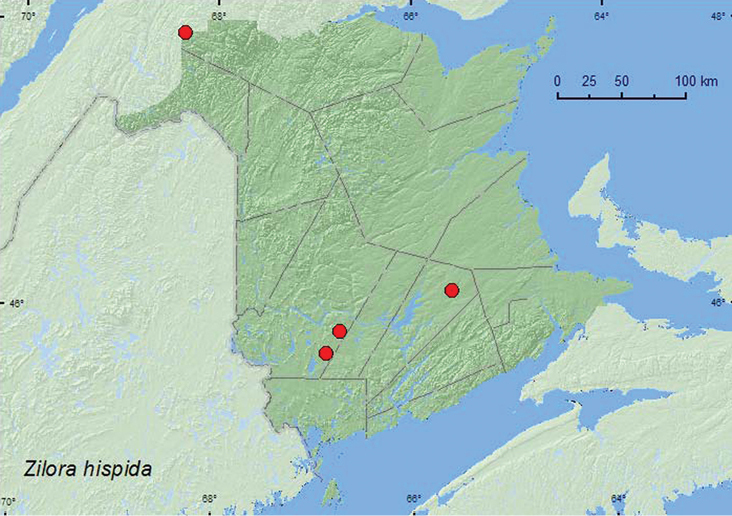
Collection localities in New Brunswick, Canada of *Zilora hispida*.

##### Collection and habitat data.

*Zilora hispida* was found in a mature and an old mixed forest, an old-growth white spruce and balsam fir forest, and an old red oak forest in New Brunswick. One adult was collected from under bark of red spruce infested with Scolytinae; four others were captured in Lindgren funnel traps. This species has been reared from *Picea glauca* in Montana (Majka and Pollock 2006) and has been found on *Abies balsamea* in Maine (Dearborn and Donahue 1993), otherwise little is known about its biology and habitat associations. Adults were captured during May, June, and August.

##### Distribution in Canada and Alaska.

YK, BC, AB, ON, QC, **NB**, NS, NF (LeSage 1991b; Majka and Pollock 2006).

#### Zilora nuda

Provancher, 1877**

http://species-id.net/wiki/Zilora_nuda

Map 25

##### Material examined.

**New Brunswick, Queens Co.**, Cranberry Lake P.N.A, 46.1125°N, 65.6075°W, 3–13.V.2011, 13–25.V.2011, M. Roy & V. Webster, old red oak forest, Lindgren funnel traps (2, AFC, RWC).

**Map 25.**
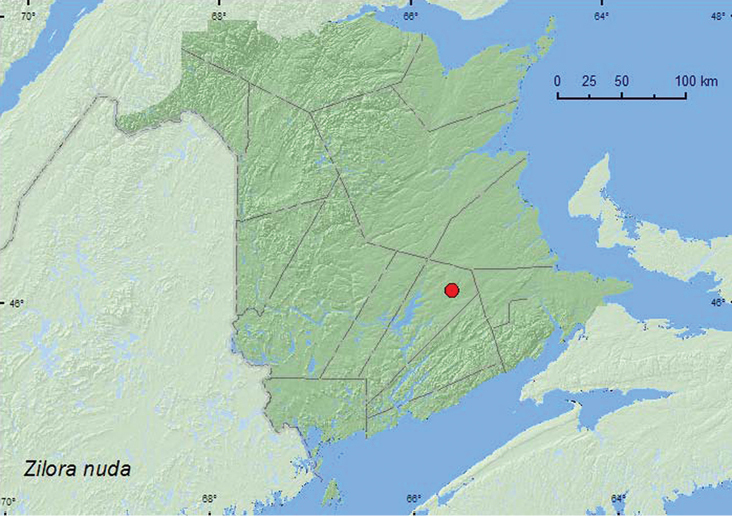
Collection localities in New Brunswick, Canada of *Zilora nuda*.

##### Collection and habitat data.

Both specimens were captured during May in Lindgren funnel traps deployed in an old red oak forest.

##### Distribution in Canada and Alaska.

QC, **NB** (LeSage 1991b).

## Supplementary Material

XML Treatment for Mycetophagus flexuosus

XML Treatment for Mycetophagus punctatus

XML Treatment for Mycetophagus serrulatus

XML Treatment for Mycetophagus pluripunctatus

XML Treatment for Mycetophagus quadriguttatus

XML Treatment for Litargus didesmus

XML Treatment for Litargus tetraspilotus

XML Treatment for Tetratoma tessellata

XML Treatment for Tetratoma variegata

XML Treatment for Pisenus humeralis

XML Treatment for Penthe obliquata

XML Treatment for Hallomenus serricornis

XML Treatment for Eustrophus tomentosus

XML Treatment for Synstrophus repandus

XML Treatment for Symphora flavicollis

XML Treatment for Symphora rugosa

XML Treatment for Microscapha clavicornis

XML Treatment for Orchesia cultriformis

XML Treatment for Orchesia ovata

XML Treatment for Enchodes sericea

XML Treatment for Scotochroides antennatus

XML Treatment for Phloeotrya fusca

XML Treatment for Spilotus quadripustulatus

XML Treatment for Zilora hispida

XML Treatment for Zilora nuda
